# Evaluation of mixed energy partial arcs for volumetric modulated arc therapy for prostate cancer

**DOI:** 10.1002/acm2.12561

**Published:** 2019-03-12

**Authors:** Shadab Momin, James L. Gräfe, Rao F. Khan

**Affiliations:** ^1^ Department of Radiation Oncology Washington University School of Medicine St. Louis MO USA; ^2^ Department of Physics Ryerson University Toronto ON Canada

**Keywords:** mixed energy VMAT, optimization, treatment planning

## Abstract

**Purpose:**

The purpose of this work was to investigate the dosimetric impact of mixed energy (6‐MV, 15‐MV) partial arcs (MEPAs) technique on prostate cancer VMAT plans.

**Methods:**

This work involved prostate only patients, planned with 79.2 Gy in 44 fractions to the planning target volume (PTV). Femoral heads, bladder, and rectum were considered organs at risk. This study was performed in two parts. For each of the 25 patients in Part 1, two single‐energy single‐arc plans, a 6 MV‐SA plan and a 15 MV‐SA plan, and a third MEPA plan involving composite of 6‐MV anterior–posterior partial arcs and a 15‐MV lateral partial arc weighted 1:2 were created. The dosimetric difference between MEPA(6/15 MV 1:2 weighted) and 6 MV‐SA plans, and MEPA(6/15 MV 1:2 weighted) and 15 MV‐SA plans were measured. In the Part 2 of this study, a second MEPAs plan (6 MV anterior–posterior arcs and 15 MV lateral arcs weighted 1:1), (MEPA 6/15 MV 1:1 weighted), was generated for 15 patients and compared only with two single‐energy partial arcs plans, a 6 and a 15 MV‐PA, to investigate the influence of the energy only. Dosimetric parameters of each structure, total monitor‐units (MUs), homogeneity index (HI), and conformity number (CN) were analyzed.

**Results:**

In Part 1, no statistically significant differences were observed for mean dose to PTV and CN for MEPAs (6/15 MV 1:2 weighted) vs 6 and 15 MV‐SA. MEPAs (6/15 MV 1:2 weighted) increased HI compared to 6 and 15 MV‐SA (*P* < 0.0005; *P* < 0.0005). MEPAs (6/15 MV 1:2 weighted) produced significantly lower mean doses to rectum, bladder, and MUs/fraction, but higher mean doses to femoral heads, compared to 6 MV‐SA (*P* < 0.0005) and 15 MV‐SA (*P* < 0.0005). The results of Part 2 of this study showed that, in comparison to 6 and 15 MV‐PA, MEPAs (6/15 MV 1:1 weighted) plans significantly improved CNs (*P* < 0.0005; *P* < 0.0005) and produced significantly lower mean doses to the rectum and bladder (*P* < 0.0005; *P* < 0.0005). While mean doses to the PTV and femoral heads of MEPAs (6/15 MV 1:1 weighted) plans were statistically comparable to 6 MV‐PA (*P* > 0.05), MEPAs (6/15 MV 1:1 weighted) increased mean doses to left (*P* = 0.04) and right (*P* = 0.04) femoral heads compared to 15 MV‐PA. MEPAs (6/15 MV 1:1 weighted) resulted in significantly lower total MUs compared to 6 MV‐PA (*P* < 0.0005) and 15 MV‐PA (*P* = 0.04).

**Conclusion:**

The study for prostate radiotherapy demonstrated that a choice of MEPAs for VMAT has the potential to minimize doses to OARs and improve dose conformity to PTV, at the expense of a moderate increase in mean dose to the femoral heads.

## 1 INTRODUCTION

The main goal of radiation therapy is to provide dose conformity to the target in four dimensions of space and time while minimizing the dose to the normal tissues and organs at risk. Early techniques used geometric field shaping alone involving blocks or multileaf collimators (MLC) to conform to the target volume. Subsequently, intensity modulated radiation therapy (IMRT) allowed modulation of fluence across the geometrically shaped field by using multiple radiation beams of nonuniform intensities. Currently, IMRT is widely practiced in clinics owing to its dosimetric advantages such as superior target dose conformity and better OARs sparing.^1^ During the last decade, volumetric modulated arc therapy (VMAT) using modulated arcs is gaining popularity due to its improved efficiency compared to IMRT. VMAT involves the simultaneous rotational movement between the linear accelerator along with varying dose rate, gantry speed, and the shaping of multileaf collimator (MLC) leaves to produce modulated fluence while the beam is on. It has been reported by a number of studies that VMAT results in improved delivery efficiency than IMRT for various types of cancer.^2^, ^3^, ^4^, ^5^, ^6^, ^7^ A comprehensive meta‐analysis on preferred technique in prostate treatment has shown that, in addition to improvement in the delivery efficiency, VMAT also protects OARs better than IMRT for prostate cancer.^8^

Both IMRT and VMAT utilize inverse planning algorithms for optimization of dose to target and OARs. A clinically available optimization software optimizes fluence map for each beam angle to achieve dose‐volume objectives. However, it does not optimize for couch angle or photon energy. The selection of these parameters depends on the tumor location and the experience of a treatment planner. The preference on selection of photon beam energy for deep seated targets varies due to various energy‐related dosimetric consequences. For instance, use of low energy photon beams (≤6 MV) generates narrow penumbra, which results in tighter dose distribution around the target. However, for deep seated targets, it may result in a higher surface dose. Higher energy photon beams, on the other hand, increase forward scattering of electrons and photons, resulting in a low skin dose, but may result in undesirable dose to the patient from secondary neutrons (especially for 18 MV). A number of previous studies for prostate cancer reported dosimetric benefits of using a higher energy photon beam over 6 MV photon beam.^9^, ^10^, ^11^, ^12^

Only a handful of studies, however, have compared dosimetric results of mixed energy (both low and high MV) IMRT plans with a single energy IMRT for deep seated targets.^12^, ^13^ While Park et al.^12^ performed a sequential optimization of photon beam energy (i.e., generation of 6 MV fluence maps followed by 15 MV fluence maps) using a commercial treatment‐planning software, McGeachy et al.^13^ performed simultaneous optimization of photon beam energy and fluence maps using an external optimizer. Nonetheless, both studies showed that mixed energy IMRT improved overall quality of the treatment plans including better sparing of OARs.

To our knowledge, for VMAT, only one study has investigated the dosimetric influence of mixed energy VMAT approach for prostate cancer.^14^ Pokharel compared the mixed energy full arcs VMAT plans (a composite of 6 MV primary plan and 16 MV boost plan) with a single‐energy full arcs VMAT plans of either low or high energy. Pokharel reported mixed energy VMAT plans to be superior over a single‐energy VMAT plans in better sparing of OARs while maintaining dose conformity to the target. Since the current commercial VMAT optimizers are not capable of optimizing a single plan with more than one energy, a mixed energy VMAT plan can only be created by combining two or more individual plans.^15^ In this work, we created mixed energy partial arcs (MEPAs) plans by manually merging a 6 MV partial arcs plan and a 15 MV partial arcs plan. To our knowledge, the investigation on the dosimetric impacts of MEPAs on VMAT plans for prostate has not been reported in the literature. The aim of this work, therefore, was to further explore the scope of using two mixed energy VMAT techniques for prostate cancer by:

- evaluating the additive effects of photon energy and dose weighting in Part 1 through dosimetric comparisons of MEPAs (6/15 MV 1:2 weighted) plans with 6 MV single‐arc (6 MV‐SA) plans and 15 MV single‐arc (15 MV‐SA) plans.
- investigating the sole effect of photon beam energy in Part 2 through dosimetric comparisons of an equal dose weighted MEPAs (6/15 MV 1:1 weighted), with 6 MV only partial arcs, (6 MV‐PA) plans, and 15 MV only partial arcs (15 MV‐PA plans) plans.

## 2 MATERIALS AND METHODS

### 2.A Patient selection

**Figure 1:**
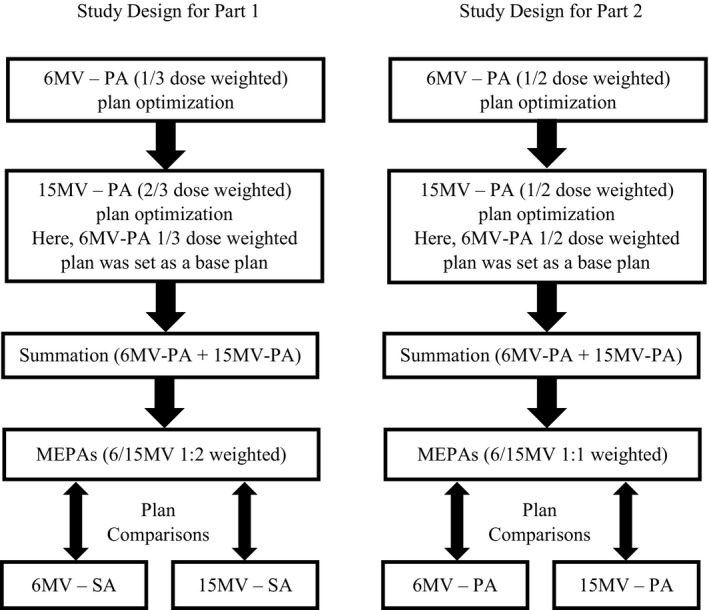
Flow charts illustrating the steps taken in generating mixed energy partial arcs plans and their comparisons with single energy plans in Part 1 (left) and Part 2 (right) of this study.

A cohort of 25 patients with intermediate risk of prostate cancer who underwent radiation therapy was randomly selected for Part 1 of this study. A subset of 15 patients was randomly selected for the Part 2 of this study. For both studies, mean and standard deviation of planning measurements such as anterior‐posterior separation, lateral separation, planning target volume (PTV), bladder, rectum, and femoral head volumes are summarized in Table 1. Figure 1 illustrates the steps taken in generating MEPAs plans and their comparisons with single energy plans in each part of the study.

**Table 1:** Summary of planning measurements for both parts of the study

Comparison	Studies	
	First part	Second part
	MEPAs(6/15 MV 1:2 weighted) vs 6 MV‐SA and 15 MV‐SA, respectively	MEPAs(6/15 MV 1:1 weighted) vs 6 MV‐PA and 15 MV‐PA, respectively
Sample size	25	15
Age (yr)	67 ± 10	71 ± 9
A‐P separation (cm)	23 ± 3	24 ± 3
Lateral separation (cm)	39 ± 6	40 ± 5
PTV volume (cc)	86 ± 25	85 ± 18
Bladder volume (cc)	251 ± 115	229 ± 96
Rectum volume (cc)	74 ± 34	81 ± 36
Right femur volume (cc)	182 ± 20	188 ± 21
Left femur volume (cc)	181 ± 21	187 ± 23

### 2.B CT simulation and contouring

Computed tomography (CT) scanning and simulations were performed using Philips Brilliance Big Bore Scanner (Philips Medical, Cambridge, MA) with patients in a supine position and by following the standard CT scan protocol. The thickness of each CT image in axial dimension was 1.5 mm. The contouring of prostate, left femur, right femur, bladder, and rectum was performed by a radiation oncologist on the axial slices of the CT using the Varian Eclipse™ treatment planning system version 13.7 (Varian Medical Systems, Palo Alto, CA). The OARs included bladder, rectum, left, and right femur. The OAR volumes were contoured according to the radiation therapy oncology group (RTOG‐0815) protocol.^16^ The prostate was defined as a clinical target volume from which the PTV was generated by adding a 5 mm margin in all directions. Mean PTV volume was 86 ± 25 cc.

### 2.C Treatment planning and optimization

In both parts of this study, the total prescription dose (PD) was 79.2 Gy in 44 fractions, with a daily dose of 180 cGy. The goal of treatment plan was to cover 95% of the PTV volume by at‐least 95% of the PD with no more than 2% of the PTV receiving 107%. The dosimetric constraints were originally derived based on the quantitative analysis of normal tissue effects (QUANTEC) requirement for prostate cancer.^17^ For OARs, the goal was to meet the clinically acceptable dose‐volume requirements as shown in Table 2.

**Table 2:** The QUANTEC based dose‐volume restrictions for OARs including femoral heads, rectum, and bladder

Femoral heads	V50 < 5%
Rectum	V75 < 15%, V70 < 20%, V65 < 25%, V60 < 35%, V50 < 50%
Bladder	V80 < 15%, V75 < 25%, V70 < 35%, V65 < 50% D_max_ < 65 Gy

Femoral heads V50 < 5% represents no more than 5% of either femoral heads should receive a dose of 50 Gy or more. D_max_ = Maximum Dose.

#### 2.C.1. Treatment Plans

For each of the 25 patients in the Part 1 of the study, three volumetric modulated arc plans were generated using the RapidArc™ module in Eclipse™: (a) 6 MV plan using a SA, (b) 15 MV plan using a SA, (c) composite plan using 6 MV anterior–posterior partial arcs, and 15 MV lateral arcs weighted 1:2 called MEPAs (6/15 MV 1:2 weighted).

The dosimetric outcome of MEPAs (6/15 MV 1:2 weighted) plans in part 1 of this study may result from additive effects of unequal dose weighting and the energy. Furthermore, RapidArc™ TPS for VMAT is an aperture/control point based optimization algorithm, which may act slightly different for single‐energy single‐arc vs single‐energy partial arcs. Therefore, to eliminate this effect in addition to unequal dose weighting, in Part 2, we performed another study with 15 patients in which MEPAs plans weighted 1:1 called MEPAs (6/15 MV 1:1 weighted) were compared with the 6 MV only partial arcs plans (6 MV‐PA) and 15 MV only partial arcs (15 MV‐PA) plans. Thus, the Part 2 of this study would essentially evaluate the influence of photon beam energy only.

#### 2.C.2. Gantry and collimator settings

**Figure 2:**
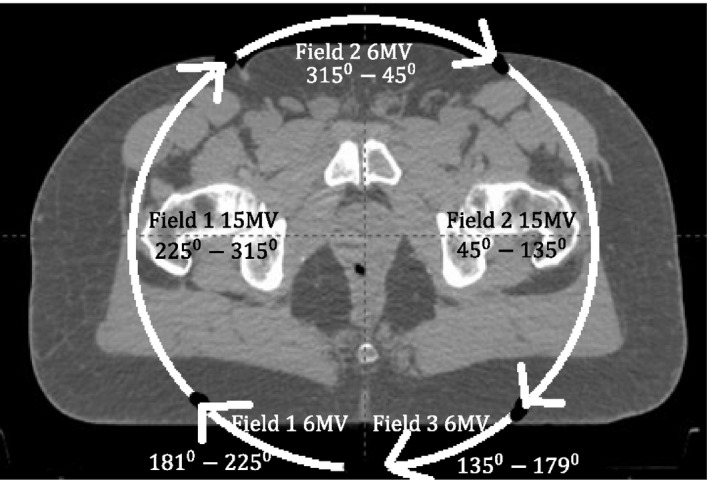
Arc start and stop angles for a Volumetric Arc Therapy (VMAT) mixed energy partial arcs plan using partial arcs in Eclipse treatment planning system.

In Part 1 of this study, the gantry angle was set to rotate clockwise from 181° to 179° for 6 MV‐SA and 15 MV‐SA plans. For MEPAs (6/15 MV 1:2 weighted), the arc start and stop angles for a 6 MV were 181°–225°, 315°–45°, and 135°–179° rotating clockwise, whereas for a 15 MV plan were 225°–315° and 45°–135° rotating clockwise (Fig. 2).

In Part 2, the arc arrangement for MEPAs (6/15 MV 1:1 weighted), 6 MV‐PA (6 MV as anterior–posterior arcs and 6 MV as lateral arcs) and 15 MV‐PA (15 MV as anterior–posterior arcs and 15 MV as lateral arcs) were same as the one for MEPAs (6/15 MV 1:2 weighted).

In both parts, the collimator angle was set to 90° for all plans as it is considered to be a good choice for better OARs sparing in prostate cancer VMAT.^18^ The isocenter was placed at the center of mass of the PTV for all the plans.

#### 2.C.3. Optimization parameters

In Part 1 of this study, two separate single‐energy single‐arc (a 6 MV‐SA plan and a 15 MV‐SA) plans were generated by setting the optimization objectives, dose volume constraints and priority weighting factors as illustrated in (Table 3). For MEPAs, the following steps were followed:

1. A 6 MV anterior and posterior partial arcs plan was created by setting the optimization objectives, constraints, and weightings as shown in (Table 3). The 6 MV partial arcs plan delivered 26.4 Gy over 44 fractions.
2. A 15 MV lateral arcs plan was then generated by setting the optimization objectives, constraints, and weightings as shown in (Table 3). Since Eclipse does not allow mixing energies in RapidArc™ module, the 15 MV lateral arcs plan was optimized by using the 6 MV anterior‐posterior arcs plan as a base plan. The 15 MV lateral arcs plan delivered 52.8 Gy over 44 fractions.
3. As a final step, plans from the previous steps, 6 MV anterior‐posterior arcs plan and 15 MV lateral arcs plan were summated to generate a MEPAs (6/15 MV 1:2 weighted) plan.

**Table 3:** Dose volume constraints and priority factors set in RapidArc^™^ treatment planning software for optimization of 25 patients in first study involving MEPA(6/15 MV 1:2 weighted), 6 MV‐SA and 15 MV‐SA plans

Structure	Vol (%)	Dose (% of PD)	Priority factor
PTV	0	105%	250
	100	101.1%	250
Bladder	0	98.5%	150
	12	50.6%	150
	38	31.2%	150
	64	23.6%	150
Rectum	0	104.0%	150
	12	59.0%	150
	39	39.6%	150
	75	14.2%	150
Left femur	0	50%	150
Right femur	0	50%	150

PD: prescribed dose; 6 MV‐SA: 79.2 Gy (PD); 15 MV‐SA: 79.2 Gy (PD); 6 MV‐partial arcs: 26.4 Gy (PD); 15 MV‐partial arcs: 79.2 Gy (PD); PTV: planning target volume.

In the Part 2 of this study, 6 MV only partial arcs plans (6 MV‐PA), 15 MV only partial arcs plans (15 MV‐PA), and MEPAs plans weighted 1:1, MEPAs (6/15 MV 1:1 weighted), were generated by using optimization parameters shown in Table 4. MEPAs (6/15 MV 1:1 weighted) plans were generated by following the aforementioned steps 2 and 3, but with an equal dose weighting.

**Table 4:** Dose volume constraints and priority factors set in RapidArc^™^ treatment planning software for optimization of 15 patients in second study involving MEPA(6/15 MV 1:1 weighted), 6 and 15 MV‐PA plans

Structure	Vol (%)	Dose (% of PD)	Priority factor
PTV	0	105%	250
	100	101.1%	250
Bladder	0	98.5%	35
	12	50.6%	35
	38	31.2%	35
	64	23.6%	35
Rectum	0	104.0%	35
	12	59.0%	35
	39	39.6%	35
	75	14.2%	35
Left femur	0	50%	35
Right femur	0	50%	35

PD: Prescribed dose; 6 MV‐PA: 79.2 Gy (PD); 15 MV‐PA: 79.2 Gy (PD); 6 MV‐partial arcs: 39.6 Gy (PD); 15 MV‐partial arcs: 79.2 Gy (PD); PTV: planning target volume.

In both parts of this study, the beam arrangement (6 MV anterior–posterior arcs and 15 MV lateral arcs) was selected based on the anatomical location of the target and surrounding OARs, and required photon beam penetrating power. The beam parameters, optimization objectives, dose‐volume constraints, and weighting factors were kept constant for the 25 patients studied in Part 1 and for the 15 patients studied in Part 2.

To avoid hot spots in the normal tissue, normal tissue objective (NTO) feature of Eclipse™ TPS was used with the priority of 100 in combination with the falloff value of 0.05 cm^−1^. The NTO distance from the target border, start dose, and end dose were 1 cm, 105%, and 60%, respectively. No normalization was required in both studies to achieve dosimetric goals of the treatment.

### 2.D Dosimetric parameters

The dose volume histograms (DVH) were generated for each plan in Eclipse for dosimetric evaluation and comparison. The dose calculation was performed with the anisotropic analytical algorithm (AAA — Version 13.7.16) with a 2.5 mm calculation grid. PTV coverage was evaluated by calculating conformity number (CN) as defined below.^19^, ^20^

(1) $${\text{CN}}_{{\mathrm{Van}}^{\prime }t\;\mathrm{Riet}}=\left(\frac{{\text{TV}}_{T,\mathrm{ref}}}{V_{\mathrm{ref}}}\right)\times \left(\frac{{\text{TV}}_{T,\mathrm{ref}}}{V_{T}}\right)$$

where TV_T,ref_. represents the volume of the target volume covered by the 95% of the isodose, V_ref_ represents the total volume receiving 95% of the isodose (V_ref_ was determined by converting isodose to structure feature in Eclipse), V_*T*_ represents PTV volume. This conformity assessment in Eq. (1) accounts for both target coverage (the first brackets) and the proximity of isodose line to the target (the second brackets). A CN value closer to 1 is considered a perfectly conformal plan.

Similarly, the mean and maximum dose, and hotspot determined by D_2%_ (dose received by 2% of PTV) were recorded for each case. To evaluate the dose homogeneity within the PTV, the homogeneity index (HI) was defined as per ICRU83 by taking a ratio of difference of D_2%_ (dose delivered to 2% of the PTV) and D_98%_ (dose delivered to 98% of the PTV), and dose delivered to 50% of the PTV.^21^ The plan is considered homogeneous if the value of HI is close to zero.

(2) $$\text{HI}=\frac{D_{2\%}-D_{98\%}}{D_{50\%}}$$

For OARs, the volumes receiving 70, 50, 30, and 20 Gy (V_70Gy_, V_50Gy_, V_30Gy_, and V_20Gy_) were calculated to evaluate various irradiated volumes of bladder and rectum. The mean dose was calculated to evaluate dose to femoral heads.

The average differences between 6 MV‐SA and MEPAs, and between 15 MV‐SA and MEPAs in corresponding dosimetric parameter were evaluated by using Eq. (3).^14^

(3) $$D_{\mathrm{avg}}^{\mathrm{QMV}}\left(x\right)=\frac{1}{n}\sum_{i=1}^{n}\left[\frac{{\left(\mathrm{QMV}\mathrm{SA}\right)}_{i}-{\left(\mathrm{MEPAs}\right)}_{i}}{{\left(\mathrm{QMV}\mathrm{SA}\right)}_{i}}\times 100\right]$$

In Eq. (3), Q represents beam energy that is, 6 or 15 MV, *x* represents the dosimetric parameter to be analyzed, and *n* represents the total number of patients, 25 for the first part, and 15 for the second part. Since current standards of care use single arc with single energy, they were compared to the MEPAs. The 6 MV‐SA plans and 15 MV‐SA plans were used as standard plans to evaluate the average difference (D_avg_) between 6 MV‐SA and MEPAs (6/15 MV 1:2 weighted), and 15 MV‐SA and MEPAs (6/15 MV 1:2 weighted). This was repeated for the Part 2 in which 6 and 15 MV‐PA plans were used as standard plans to evaluate the average difference between 6 MV‐PA and MEPAs (6/15 MV 1:1 weighted), and 15 MV‐PA and MEPAs (6/15 MV 1:1 weighted).

### 2.E Statistical analysis

In Part 1, the dosimetric parameters of MEPAs (6/15 MV 1:2 weighted) plans were statistically compared with the dosimetric parameters of 6 and 15 MV‐SA using a two‐tailed paired‐sample *t*‐test. In addition, the 95% confidence interval is included for each *P*‐value.

In Part 2, the dosimetric parameters of MEPAs (6/15 MV 1:1 weighted) plans were statistically compared with the dosimetric parameters of 6 and 15 MV‐PA using a two‐tailed paired‐sample *t*‐test.

At this point, it is important to note that both parts of this study are independent of each other and no cross comparison was done between dosimetric parameters of the two parts. Statistical analysis was conducted by using IBM SPSS Statistics 24 (IBM Corp. Released 2017. IBM SPSS Statistics for Windows, Version 24.0. Armonk, NY: IBM Corp). For both studies, a *P* < 0.05 was considered to be statistically significant. Prior to two‐tailed *t*‐test, the data were checked for normal distribution by performing the Shapiro–Wilk test.^22^

## 3 RESULTS

### 3.A Part 1

#### 3.A.1 Dosimetry

The dosimetric parameters averaged over 25 cases for the 6 MV‐SA, 15 MV‐SA, and MEPAs (6/15 MV 1:2 weighted) is highlighted in Table 5. The statistical differences between 6 MV‐SA and MEPAs (6/15 MV 1:2 weighted), and 15MV‐SA and MEPAs (6/15 MV 1:2 weighted) plans are shown in Table 6. The average differences, $D_{\mathrm{avg}}^{6\mathrm{MV}}{\text{and}\;}_{\mathrm{avg}}^{15\mathrm{MV}}$, for dosimetric parameters of the PTV, bladder, rectum, and as well as number of Monitor Units (MU), CI, and HI are shown in Table 7.

**Table 5:** The dosimetric parameters for 6 MV‐SA, 15 MV‐SA, and MEPAs (6/15 MV 1:2 weighted) plans. The data are averaged over the cohort of 25 patients

Structure	Dosimetric parameter	Avg. ± SD 6 MV‐SA	Avg. ± SD 15 MV‐SA	Avg. ± SD MEPAs
PTV	Max dose (Gy)	87.1 ± 1.2	86.6 ± 1.3	86.0 ± 1.2
	95% CI (Gy)	86.6–87.6	86.0–87.1	85.2–86.7
	Mean dose (Gy)	81.1 ± 0.4	81.3 ± 0.6	81.2 ± 0.4
	95% CI (Gy)	80.9–81.3	81.1–81.6	81.0–81.4
	D_2%_ (Gy)	83.9 ± 0.6	84.0 ± 0.7	84.0 ± 0.6
	95% CI (Gy)	86.7–84.2	83.7–84.3	83.7–84.3
	HI	0.08 ± 0.02	0.08 ± 0.02	0.09 ± 0.02
	95% CI	0.08–0.09	0.08–0.09	0.09–0.1
	CN	0.82 ± 0.04	0.82 ± 0.04	0.83 ± 0.05
	95% CI	0.80–0.83	0.80–0.84	0.80–0.84
Bladder	Max dose (Gy)	85.9 ± 1.7	85.5 ± 1.6	84.6 ± 1.2
	95% CI (Gy)	85.3–86.6	84.9–86.3	84.1–85.1
	Mean dose (Gy)	14.2 ± 6.6	14.1 ± 6.8	13.6 ± 6.1
	95% CI (Gy)	11.5–16.9	11.3–16.9	10.8–15.8
	V_70Gy_ (%)	4.2 ± 2.0	4.2 ± 2.0	4.1 ± 2.1
	95% CI (%)	3.4–5.0	3.4–5.0	3.2–4.9
	V_50Gy_ (%)	8.9 ± 4.1	9.1 ± 4.4	8.5 ± 4.2
	95% CI (%)	7.2–10.6	7.2–10.9	6.8–10.3
	V_30Gy_ (%)	17.4 ± 9.7	17.6 ± 9.9	15.7 ± 8.8
	95% CI (%)	13.3–21.4	13.5–21.7	12.1–19.3
	V_20Gy_ (%)	22.9 ± 13.9	23.7 ± 13.8	21.2 ± 12.0
	95% CI (%)	17.1–28.6	18.0–29.3	16.2–26.1
Rectum	Max dose (Gy)	85.0 ± 1.7	84.8 ± 1.4	84.1 ± 1.4
	95% CI (Gy)	84.3–85.7	84.3–85.4	83.5–84.6
	Mean dose (Gy)	25.3 ± 3.4	25.7 ± 3.8	23.0 ± 3.6
	95% CI (Gy)	23.9–26.7	24.1–27.3	21.5–24.5
	V_70Gy_ (%)	6.6 ± 2.1	6.7 ± 2.1	7.1 ± 2.1
	95% CI (%)	5.7–7.4	5.8–7.5	6.2–7.9
	V_50Gy_ (%)	15.2 ± 2.9	16.3 ± 3.5	15.8 ± 3.8
	95% CI (%)	14.0–16.4	14.8–17.7	14.3–17.4
	V_30Gy_ (%)	40.1 ± 5.8	40.1 ± 7.1	31.4 ± 6.8
	95% CI (%)	37.7–42.5	37.5–43.3	28.6–34.2
	V_20Gy_ (%)	52.2 ± 8.0	53.2 ± 8.6	42.4 ± 7.2
	95% CI (%)	48.9–55.5	49.6–56.7	39.4–45.4
L femur	Mean dose (Gy)	11.1 ± 2.2	11.0 ± 2.2	14.9 ± 3.1
	95% CI (Gy)	10.2–12.0	10.1–12.0	13.6–16.2
	Max dose (Gy)	30.7 ± 5.6	30.0 ± 5.2	39.6 ± 4.7
	95% CI (Gy)	28.5–32.9	28.0–32.0	37.7–41.4
R femur	Mean dose (Gy)	10.9 ± 2.9	11.0 ± 2.3	15.3 ± 2.9
	95% CI (Gy)	9.8–12.2	10.0–12.1	14.1–16.5
	Max dose (Gy)	30.9 ± 5.4	30.6 ± 5.6	40.5 ± 3.5
	95% CI (Gy)	28.8–33.0	28.4–32.8	39.2–41.9
MUs		637 ± 84	514 ± 50	435 ± 104
	95% CI (MUs)	602–673	493–535	398–474

MEPAs: mixed energy partial arcs; SA: single arc; PTV: planning target volume; SD: standard deviation.

**Table 6:** Statistical comparison of dosimetric parameters between (a) 6 MV‐SA and MEPAs (6/15 MV 1:2 weighted) and (b) 15 MV‐SA and MEPAs (6/15 MV 1:2 weighted). The dosimetric parameters are averaged over the cohort of 25 patients

Structure	Dosimetric parameter	6MV‐SA vs MEPAs (1:2 weighted)		15MV‐SA vs MEPAs (1:2 weighted)	
		*P*‐value	95% CI	*P*‐value	95% CI
PTV	Max dose (Gy)	<0.0005	0.70, 1.59	0.03	0.07, 1.23
	Mean dose (Gy)	0.01	−0.24, −0.03	0.06	−0.01, 0.24
	D_2%_ (Gy)	0.67	−0.26, 0.17	0.87	−0.18, 0.21
	HI	<0.0005	−0.02, −0.01	<0.0005	−0.02, −0.005
	CN	0.1	−0.02, 0.002	0.1	−0.02, 0.003
Bladder	Max dose (Gy)	0.01	0.67, 2.00	<0.0005	0.21, 1.66
	Mean dose (Gy)	<0.0005	0.49, 1.41	<0.0005	0.36, 1.36
	V_70Gy_ (%)	0.08	−0.02, 0.27	0.04	0.01, 0.27
	V_50Gy_ (%)	0.09	−0.06, 0.74	0.02	0.10, 0.97
	V_30Gy_ (%)	0.002	0.70, 2.65	<0.0005	1.01, 4.34
	V_20Gy_ (%)	0.02	0.21, 3.27	0.001	1.11, 3.87
Rectum	Max dose (Gy)	0.001	0.42, 1.44	0.002	0.31, 1.18
	Mean dose (Gy)	<0.0005	1.41, 3.08	<0.0005	1.74, 5.98
	V_70Gy_ (%)	0.005	−0.88, −0.18	0.01	−0.75, −0.10
	V_50Gy_ (%)	0.22	−1.61, 0.38	0.39	−0.62, 1.54
	V_30Gy_ (%)	<0.0005	6.52, 10.84	<0.0005	6.26, 11.7
	V_20Gy_ (%)	<0.0005	6.84, 12.72	<0.0005	7.72, 13.79
Left femur	Mean dose (Gy)	<0.0005	−4.77, ‐2.79	<0.0005	−4.94, −2.78
	Max dose (Gy)	<0.0005	−11.02, −6.78	<0.0005	−11.96, −7.19
Right femur	Mean dose (Gy)	<0.0005	−5.53, −3.04	<0.0005	−5.43, −3.04
	Max dose (Gy)	<0.0005	−11.53, −7.72	<0.0005	−11.83, −8.00
MUs		<0.0005	163.4, 239.6	<0.0005	43.14, 113.3

MEPAs: mixed energy partial arcs; SA: single arc; MUs: monitor units; Avg: average; PTV: planning target volume; SD: standard deviation; *P* ≤ 0.0005 represents a *P* value of 0.

**Table 7:** The average difference, D_avg_ (%), of dosimetric parameters between 6 MV‐SA and MEPAs (6/15 MV 1:2 weighted), and between 15 MV‐SA and MEPAs (6/15 MV 1:2 weighted)

Structure	Dosimetric parameter	Avg_diff_ ± SD. 6 MV‐SA vs MEPAs $\left(D_{\mathrm{Avg}}^{6\mathrm{MV}}\right)$	Avg_diff_. ± SD 15 MV‐SA vs MEPAs $\left(D_{\mathrm{Avg}}^{15\mathrm{MV}}\right)$
PTV	Min dose (%)	0 ± 4.0	1.0 ± 3.59
	Max dose (%)	0.7 ± 3.3	0.9 ± 1.84
	Mean dose (%)	‐0.9 ± 3.6	0.3 ± 0.99
	HI (%)	−22.4 ± 24.4	−19.0 ± 25.7
	CN (%)	−1.3 ± 3.5	−1.1 ± 3.5
Bladder	Max dose (%)	1.6 ± 1.9	1.1 ± 2.0
	Mean dose (%)	6.2 ± 7.0	4.7 ± 7.1
	V_70Gy_ (%)	5.8 ± 13.7	5.7 ± 11.1
	V_50Gy_ (%)	5.3 ± 14.7	6.5 ± 13.0
	V_30Gy_ (%)	8.7 ± 13.2	10.2 ± 12.0
	V_20Gy_ (%)	5.3 ± 11.8	9.3 ± 10.7
Rectum	Max dose (%)	1.1 ± 1.4	0.9 ± 1.2
	Mean dose (%)	8.8 ± 7.7	10.1 ± 8.2
	V_70Gy_ (%)	−9.3 ± 17.1	−7.5 ± 14.6
	V_50Gy_ (%)	−4.0 ± 17.1	2.5 ± 15.7
	V_30Gy_ (%)	21.7 ± 12.9	21.6 ± 14.1
	V_20Gy_ (%)	18.2 ± 11.5	19.5 ± 11.4
L Femur	Mean dose (%)	−35.5 ± 23.1	−37.4 ± 26.5
	Max dose (%)	−31.9 ± 21.7	−34.6 ± 21.8
R Femur	Mean dose (%)	−90.6 ± 277.0	−46.4 ± 24.7
	Max dose (%)	−34.2 ± 21.4	−35.4 ± 20.6
MUs		29.8 ± 19.7	12.9 ± 26.6

V_nGy_, in terms of data, represents the percentage of structure volume receiving n Gy or more. $D_{\mathrm{Avg}}^{6\mathrm{MV}}$ and $D_{\mathrm{Avg}}^{15\mathrm{MV}}$ were calculated using Eq. (3). PTV: planning target volume

#### 3.A.2 Doses to the PTV

Mixed energy partial arcs (6/15 MV 1:2 weighted) resulted in a lower maximum dose to the PTV in comparison to 6 MV‐SA (*P* < 0.0005) and 15 MV‐SA (*P* < 0.0005). **(**Table 5 **)**. Mean doses to the PTV of MEPAs (6/15 MV 1:2 weighted) plans was comparable to 15 MV‐SA plans (*P* = 0.06), but higher compared to 6 MV‐SA (*P* = 0.01) plans (Tables 5 and 6). The D_2%_ of the PTV of MEPAs (6/15 MV 1:2 weighted) plans was comparable to both 6 MV‐SA (*P* = 0.67) and 15 MV‐SA (*P* = 0.87) plans (Table 6).

In comparison to 6 and 15 MV‐SA plans, MEPAs (6/15 MV 1:2 weighted) produced statistically equivalent conformity number (*P* = 0.1), however, it resulted in slightly inferior target homogeneity index (*P* < 0.0005) (Table 6). A negative average difference$D_{\mathrm{avg}}^{6\mathrm{MV}}{\text{and}\;}_{\mathrm{avg}}^{15\mathrm{MV}}$, indicated higher values for HI of MEPAs (6/15MV 1:2 weighted) plans (Table 7).

#### 3.A.3 Doses to the bladder

As indicated by positive values of $D_{\mathrm{avg}}^{6\mathrm{MV}}{\text{and}\;}_{\mathrm{avg}}^{15\mathrm{MV}}$ in Table 7, the dosimetric parameters for bladder were always lower for MEPAs (6/15 MV 1:2 weighted) plans compared to 6 and 15 MV‐SA plans (Tables 5 and 7). MEPAs (6/15 MV 1:2 weighted) irradiated significantly lower volume than 6 and 15 MV‐SA, with an exception of V_70Gy_ (*P* = 0.08) and V_50Gy_ (*P* = 0.09) for 6 MV‐ SA (Tables 6 and 7). This can also be observed in 95% CI for each *P*‐value, which excludes the null value, zero, for significance and includes the null value, zero, for insignificance. Furthermore, the maximum dose to bladder exceeded 65 Gy for all three techniques without significant difference among three techniques. (Tables 5 and 6).

#### 3.A.4 Doses to the rectum

The mean dose to the rectum was ~2 Gy lower for MEPAs (6/15 MV 1:2 weighted) plans compared to 6 MV‐SA (*P* < 0.0005) and 15 MV‐SA (*P* < 0.0005) plans (Tables 5 and 6) with a positive average difference $D_{\mathrm{avg}}^{6\mathrm{MV}}{\text{and}\;}_{\mathrm{avg}}^{15\mathrm{MV}}$ of 9 ± 8% and 10 ± 8% (Table 7). MEPAs (6/15 MV 1:2 weighted) plans covered significantly lower volume of rectum at V_30Gy_ and V_20Gy_ dose levels compared to 6 MV‐SA (*P* < 0.0005), and 15 MV‐SA (*P* < 0.0005), but not at statistical significance threshold for V_50Gy_ (Tables 5 and 6).

#### 3.A.5 Doses to the femoral heads

MEPAs (6/15 MV 1:2 weighted) resulted in an increased mean doses and maximum doses to both femoral heads by ~4.0 and ~10.0 Gy compared to 6 MV‐SA (*P* < 0.0005) and 15 MV‐SA (*P* < 0.0005; Tables 5 and 6). This difference can be observed by negative values of $D_{\mathrm{avg}}^{6\mathrm{MV}}{\text{and}\;}_{\mathrm{avg}}^{15\mathrm{MV}}$ in **(Table** 7 **).**

#### 3.A.6 Monitor units

The number of MUs was lower for MEPAs (6/15 MV 1:2 weighted) plans by 202 and 79 MU compared to 6 MV‐SA (*P* < 0.0005) and 15 MV‐SA (*P* < 0.0005), respectively, (Tables 5 and 6).

#### 3.A.7 Dose distribution

**Figure 3:**
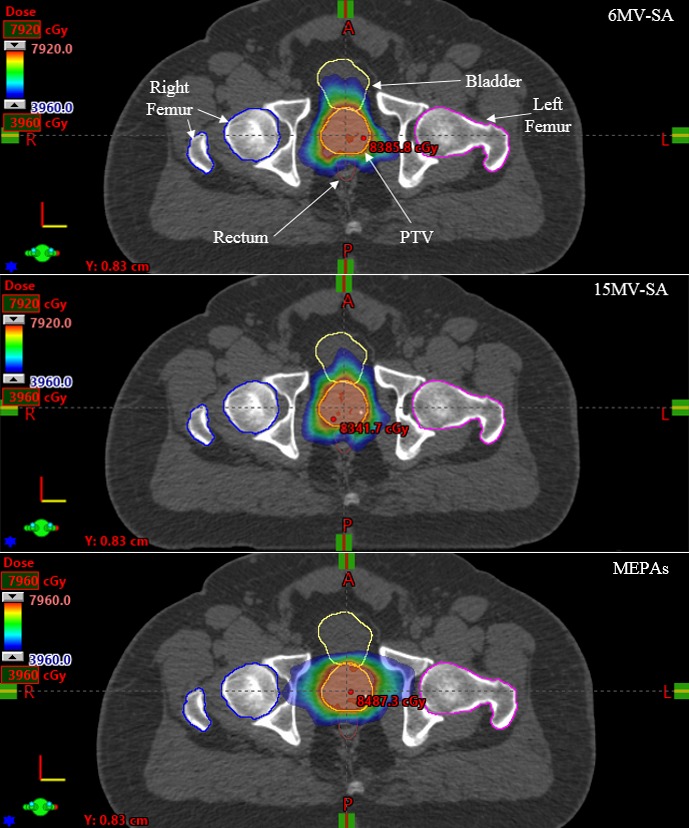
The dose distribution of a 6 MV‐SA, a 15 MV‐SA, and a MEPAs (6/15 MV 1:2 weighted) plan with an equal dose weight for one representative case in transversal views.

**Figure 4:**
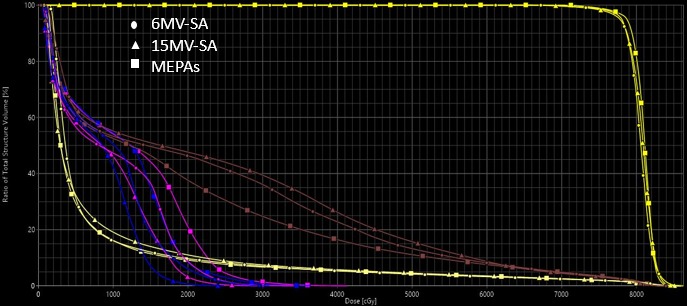
Dose volume histograms for 6 MV‐SA (Circles), 15 MV‐SA (Triangles), and MEPAs (6/15 MV 1:2 weighted) (Squares) for PTV in yellow, rectum in brown, right femur in blue, left femur in pink, and bladder in clover‐lime.

The dose distributions in color‐wash view resulting from RapidArc™ planning with 6 MV‐SA, 15 MV‐SA, and MEPAs (6/15 MV 1:2 weighted) for one representative case in transverse plane is demonstrated in Fig. 3. MEPAs (6/15 MV 1:2 weighted) technique produced tighter dose distribution in anterior‐posterior direction, where bladder and rectum are close to the PTV, but produced wider dose spread in lateral direction compared to 6 and 15 MV‐SA (Figure 3). The DVHs for all three, a 6 MV‐SA, a 15 MV‐SA, and a MEPAs (6/15 MV 1:2 weighted), plans are shown in Fig. 4, which shows large differences in the volumetric doses to rectum and femoral heads among three techniques.

### 3.B Part 2

In this part of the study, MEPAs (6/15 MV 1:1 weighted) plans were compared with 6 MV only partial arcs (6 MV‐PA) and 15 MV only partial arcs (15 MV‐PA) for a cohort of 15 patients to evaluate the influence of photon beam energy only.

#### 3.B.1 Doses to the PTV

No statistically significant differences were observed between MEPAs (6/15 MV 1:1 weighted) and 6 MV‐PA, and MEPAs (6/15 MV 1:1 weighted) and 15 MV‐PA for maximum doses to the PTV (Table 9). The mean doses to the PTV were statistically lower for MEPAs (6/15 MV 1:1 weighted) plans compared to 15 MV‐PA plans (*P* < 0.0005; Tables 8 and 9), but no statistical significance was reached for mean doses to the PTV for comparison between MEPAs (6/15 MV 1:1 weighted) plans and 6 MV‐PA plans (Table 9).

**Table 8:** The dosimetric parameters for 6 MV‐PA, 15 MV‐PA, and MEPAs (6/15 MV 1:1 weighted). The dosimetric parameters are averaged over the cohort of 15 patients

Structure	Dosimetric parameter	Avg ± SD. 6 MV‐PA	Avg. ± SD 15 MV‐PA	Avg. ± SD MEPAs
PTV	Max dose (Gy)	86.7 ± 0.9	86.0 ± 0.5	86.4 ± 1.2
	95% CI (Gy)	86.3–87.3	86.7–86.3	85.8–87.1
	Mean dose (Gy)	81.7 ± 0.4	82.1 ± 0.5	81.6 ± 0.3
	95% CI (Gy)	81.4–81.9	81.8–82.4	81.4–81.8
	D_2%_ (Gy)	84.0 ± 0.4	84.2 ± 0.5	83.7 ± 0.4
	95% CI (Gy)	83.8–84.2	83.9–84.5	83.5–84.0
	HI	0.06 ± 0.01	0.07 ± 0.01	0.07 ± 0.1
	95% CI	0.06–0.07	0.06–0.07	0.07–0.08
	CN	0.77 ± 0.05	0.78 ± 0.05	0.83 ± 0.03
	95% CI	0.75–0.80	0.75–0.81	0.81–0.84
Bladder	Max dose (Gy)	85.7 ± 1.1	85.6 ± 1.1	85.6 ± 1.6
	95% CI (Gy)	85.1–86.3	85.0–86.3	84.9–86.6
	Mean dose (Gy)	17.5 ± 8.1	17.7 ± 7.8	15.2 ± 7.6
	95% CI (Gy)	13.0–22.0	13.0–22.0	11.0–19.4
	V_70Gy_ (%)	5.0 ± 2.8	4.9 ± 2.6	4.2 ± 2.4
	95% CI (%)	3.4–6.5	3.5–6.4	2.9–5.6
	V_60Gy_ (%)	7.8 ± 4.2	7.7 ± 4.1	6.7 ± 3.7
	95% CI (%)	5.4–10.1	5.6–10.1	4.7–8.8
	V_45Gy_ (%)	13.2 ± 8.5	13.3 ± 7.9	11.1 ± 6.8
	95% CI (%)	8.5–17.9	8.9–17.7	7.3–14.9
	V_15Gy_ (%)	32.1 ± 19.9	33.9 ± 19.6	29.3 ± 19.2
	95% CI (%)	21.02–43.1	23.0–44.8	18.6–40.0
Rectum	Max dose (Gy)	85.2 ± 1.4	84.9 ± 1.1	84.7 ± 1.5
	95% CI (Gy)	84.4–86.0	84.3–85.5	83.9–85.6
	Mean dose (Gy)	31.7 ± 4.4	32.4 ± 4.2	27.2 ± 4.8
	95% CI (Gy)	29.4–34.2	30.1–34.7	24.6–30.0
	V_70Gy_ (%)	12.3 ± 5.9	11.7 ± 5.4	8.5 ± 3.6
	95% CI (%)	9.0–15.6	8.7–14.7	6.5–10.5
	V_60Gy_ (%)	19.9 ± 8.4	19.4 ± 7.9	13.8 ± 4.2
	95% CI (%)	15.2–24.6	15.1–23.8	11.5–16.2
	V_45Gy_ (%)	33.4 ± 9.9	32.3 ± 8.7	26.2 ± 5.1
	95% CI (%)	28.0–38.9	28.2–37.8	23.4–29.1
	V_15Gy_ (%)	59.3 ± 10.8	60.4 ± 11.2	56.7 ± 11.3
	95% CI (%)	53.3–65.3	54.2–66.6	50.5–63.0
Left femur	Mean dose (Gy)	10.3 ± 2.9	10.1 ± 3.0	12.0 ± 2.7
	95% CI (Gy)	8.8–11.9	8.4–11.8	10.6–13.5
Right femur	Mean dose (Gy)	10.3 ± 2.4	10.0 ± 2.8	12.3 ± 2.9
	95% CI (Gy)	8.9–11.6	8.5–11.5	10.7–13.9
MUs		553 ± 88	442 ± 57	480 ± 73
	95% CI (MUs)	504–602	411–474	440–521

MEPAs: mixed energy partial arcs; PA: partial arc; PTV: planning target volume SD: standard deviation.

**Table 9:** Statistical comparison of dosimetric parameters between (a) 6 MV‐PA and MEPAs (6/15 MV 1:1 weighted) and (b) 15 MV‐PA and MEPAs (6/15 MV 1:1 weighted) plans. The dosimetric parameters are averaged over the cohort of 15 patients

Structure	Dosimetric parameter	6 MV‐PA vs MEPAs (1:1 weighted)		15 MV‐PA vs MEPAs (1:1 weighted)	
		*P*‐value	95% CI	*P*‐value	95% CI
PTV	Max dose (Gy)	0.34	−4.40, 1.19	0.16	−1.12, 0.21
	Mean dose (Gy)	0.13	−0.04, 0.25	<0.0005	0.11, 0.3
	D_2%_ (Gy)	0.02	0.05, 0.50	0.002	0.21, 0.77
	HI	0.01	−0.01, −0.003	0.05	−0.006, −0.03
	CN	<0.0005	−0.07, −0.03	<0.0005	−0.008, 0.002
Bladder	Max dose (Gy)	0.88	−0.63, 1.52	0.99	−0.64, 0.87
	Mean dose (Gy)	<0.0005	2.1, 5.93	<0.0005	2.72, 6.59
	V_70Gy_ (%)	0.001	0.34, 1.06	<0.0005	0.44, 0.89
	V_60Gy_ (%)	0.001	0.51, 1.62	<0.0005	0.67, 1.63
	V_45Gy_ (%)	0.007	0.67, 3.57	0.001	1.16, 3.28
	V_15Gy_ (%)	0.001	1.34, 4.42	0.001	2.27, 6.86
Rectum	Max dose (Gy)	0.75	−0.56, 0.65	0.39	−0.87, 0.86
	Mean dose (Gy)	<0.0005	1.37, 3.1	<0.0005	1.53, 2.92
	V_70Gy_ (%)	0.001	1.93, 5.7	0.001	1.62, 4.71
	V_60Gy_ (%)	0.001	2.96, 9.19	0.001	1.29, 2.81
	V_45Gy_ (%)	0.007	2.37, 12.06	0.003	2.77, 10.74
	V_15Gy_ (%)	0.01	1.29, 3.92	<0.0005	2.41, 5.01
Left femur	Mean dose (Gy)	0.12	−3.83, 0.46	0.048	−3.8, −0.01
Right femur	Mean dose (Gy)	0.05	−3.98, 0.01	0.04	−4.36, −0.18
MUs		<0.0005	44, 100	0.04	−74, −2

MEPAs: mixed energy partial arcs; SA: single arc; MUs: monitor units; Avg: average; PTV: planning target volume; SD: standard deviation; *P* ≤ 0.0005 represents a *P* value of 0.

MEPAs (6/15 MV 1:1 weighted) plans significantly improved the dose conformity to the PTV compared to 6 MV‐PA (0.83 vs 0.77; *P* < 0.0005) plans and 15 MV‐PA (0.83 vs 0.78; *P* < 0.0005) plans (Table 9). This can also be observed by negative average differences, $D_{\mathrm{avg}}^{6\mathrm{MV}}{\text{and}\;}_{\mathrm{avg}}^{15\mathrm{MV}}$, in Table 10. However, MEPAs (6/15 MV 1:1 weighted) plans produced inferior target homogeneity compared to 6 MV‐PA plans (0.06 vs 0.07; *P* = 0.01) (Table 9).

**Table 10:** The average difference, D_avg_ (%), of dosimetric parameters between 6 MV‐PA and MEPAs (6/15 MV 1:1 weighted), and between 15 MV‐PA and MEPAs (6/15 MV 1:1 weighted)

Structure	Dosimetric parameter	Avg_diff._ ± SD 6 MV‐PA vs MEPAs ($D_{\mathrm{Avg}}^{6\mathrm{MV}}$)	Avg_diff._ ± SD 15 MV‐PA vs MEPA ($D_{\mathrm{Avg}}^{15\mathrm{MV}}$)
PTV	Min dose (%)	2.4 ± 3.0	2.7 ± 2.6
	Max dose (%)	0.4 ± 1.7	−0.5 ± 1.4
	Mean dose (%)	0.1 ± 0.3	0.7 ± 0.5
	HI (%)	−11.2 ± 11.0	−6.9 ± 10.0
	CN (%)	−6.8 ± 4.8	−6.4 ± 4.7
Bladder	Max dose (%)	0.1 ± 1.3	0 ± 1.8
	Mean dose (%)	14.0 ± 7.9	14.0 ± 7.6
	V_70Gy_ (%)	15.6 ± 13.7	15.9 ± 11.4
	V_60Gy_ (%)	14.7 ± 11.8	16.1 ± 12.1
	V_45Gy_ (%)	15.9 ± 11.3	18.1 ± 10.3
	V_15Gy_ (%)	10.6 ± 9.0	15.7 ± 12.1
Rectum	Max dose (%)	0.5 ± 2.2	0.1 ± 1.6
	Mean dose (%)	12.11 ± 9.0	13.9 ± 9.1
	V_70Gy_ (%)	26.6 ± 19.5	23.8 ± 18.8
	V_60Gy_ (%)	24.2 ± 21.5	23.7 ± 19.9
	V_45Gy_ (%)	17.3 ± 19.4	17.1 ± 18.1
	V_15Gy_ (%)	4.5 ± 3.7	6.3 ± 3.7
Left femur	Mean dose (%)	−26.0 ± 55.7	−26.1 ± 43.1
Right femur	Mean dose (%)	−24.7 ± 40.1	−30.0 ± 43.5
MUs		12.6 ± 8.4	−9.4 ± 15.0

V_nGy_, in terms of data, represents the percentage of structure volume receiving n Gy or more. $D_{\mathrm{Avg}}^{6\mathrm{MV}}$ and $D_{\mathrm{Avg}}^{15\mathrm{MV}}$ were calculated using Eq. (3). PTV: planning target volume.

#### 3.B.2 Doses to the Bladder

All the dosimetric parameters, except maximum dose to bladder, were statistically lower for MEPAs (6/15 MV 1:1 weighted) plans compared to 6 and 15 MV‐PA plans (Table 8 and 9). This difference can also be observed in Table 10 by positive values of average difference, $D_{\mathrm{avg}}^{6\mathrm{MV}}\;\text{and}\;D_{\mathrm{avg}}^{15\mathrm{MV}}$, for both comparisons.

#### 3.B.3 Doses to the rectum

Mean dose to the rectum for MEPAs (6/15 MV 1:1 weighted) plans was ~4 Gy lower than 6 MV‐PA (*P* < 0.0005) and 15 MV‐PA plans (*P* < 0.0005; Tables 8 and 9). Furthermore, MEPAs (6/15 MV 1:1 weighted) covered significantly lower amount of rectal volume at all dose levels (V_70Gy_ = 9%_,_ V_60Gy_ = 14%, V_45Gy_ = 26% and V_15Gy_ = 57%) compared to 6 MV‐PA (V_70Gy_ = 12%_,_ V_60Gy_ = 20%, V_45Gy_ = 33% and V_15Gy_ = 59%) and 15MV‐PA (V_70Gy_ = 12%_,_ V_60Gy_ = 19%, V_45Gy_ = 32% and V_15Gy_ = 60%; Tables 8 and 9).

#### 3.B.4 Doses to the femoral heads

MEPAs (6/15 MV 1:1 weighted) resulted in increased mean doses to right femur (*P* = 0.04) and left femur (*P* = 0.048) compared to 15 MV‐PA (Table 8 and 9). There is a noticeable difference in mean doses to femoral heads of MEPAs (6/15 MV 1:1 weighted) plans and 6 MV‐PA plans (Table 8), but no statistical significance was observed (*P* = 0.12 and *P* = 0.05 for left and right femur, respectively; Table 9).

#### 3.B.5 Monitor units

The total number of monitor units for MEPAs (6/15 MV 1:1 weighted) plans was higher than that of 15 MV‐PA plans (480 vs 442 MUs; *P* = 0.04; Tables 8 and 9) with an average negative difference of 9% (Table 10), but lower than that of 6 MV‐PA plans (480 vs 553 MUs; *P* < 0.0005; Tables 8 and 9) with an average positive difference of 13% (Table 10)**.**

#### 3.B.6 Dose distribution

**Figure 5:**
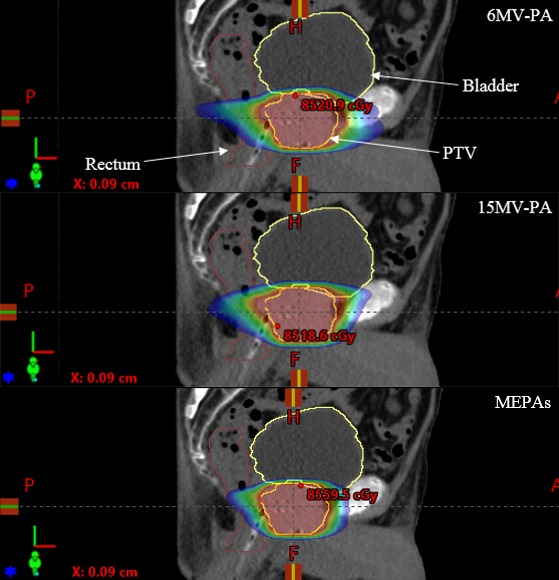
The dose distribution of a 6 MV‐PA, a 15 MV‐PA, and a MEPA (6/15 MV 1:1 weighted) plan for one representative case in sagittal views.

Figure 5 shows the dose distributions in color‐wash view for 6 MV‐PA, 15 MV‐PA, and MEPAs (6/15 MV 1:1 weighted) plans for one representative case along sagittal views. MEPAs (6/15 MV 1:1 weighted) plans appear to produce a tighter dose distribution with the greater avoidance of OARs in comparison to 6 and 15 MV‐PA plans (Figure 5).

## 4 DISCUSSION

Manually choosing multiple photon energies in an IMRT plan is not practiced very commonly except for a few clinical sites such as breast. However, for VMAT, only a single energy approach is currently being used in the clinics, presumably due to lack of sufficient evidences indicating superiority of using multiple energies over a single energy, and complexity arising due to several energies. In this work, we investigated the dosimetric quality of two MEPAs techniques for prostate cancer VMAT. In Part 1, MEPAs (6/15 MV 1:2 weighted) plans reduced the total number of monitor units, while sparing OARs and maintaining dose conformity to the PTV compared to standard 6 MV‐SA or 15 MV‐SA techniques. However, higher doses to femoral heads and slightly inferior HI of MEPAs (6/15 MV 1:2 weighted) plans should also be noted. Slightly degraded HI could be due to the optimization parameters including priority weighting factors, which, in this work, were kept the same to avoid biasing the results. Greater emphasis on priority weighting factor can essentially improve the HI. Lower doses to the bladder and rectum, and higher doses to the femoral heads by MEPAs (6/15 MV 1:2 weighted) plans were likely to be the result of 6/15 MV 1:2 dose weighting. As a result, a smaller dose proportion of PD by a lower energy (6 MV) beam produced tighter dose distribution in anterior and posterior regions of the PTV and a greater dose proportion of PD by a higher energy (15 MV) beam produced greater dose spread in the lateral direction (Figure 3). All three plans (MEPAs [6/15 MV 1:2 weighted], 6 and 15 MV‐SA) met the QUANTEC criteria, except maximum dose to bladder, due to not including the maximum dose constraint during optimization.

With an exception of degraded HI and lower MUs, the results of Part 1 of this study are in agreement with a previous study,^14^ which compared dosimetric quality of single‐energy partial‐arc (30°–165° and 195°–330°) VMAT plans with that of a single‐energy full‐arc (0°–359°) VMAT plans for prostate and demonstrated that partial arcs technique results in lower doses to the bladder and rectum but at an expense of higher doses to femoral heads.^14^ Our results, however, cannot be directly compared against the previous studies, as to our knowledge, MEPA VMAT technique has not been reported previously. A study comparing single‐arc vs dual arcs VMAT for prostate cancer demonstrated superior OARs sparing using dual arcs technique,^23^ whereas another study suggested single‐arc technique to be superior over dual arcs VMAT for OARs sparing.^17^

In Part 2 of this study, we investigated the sole effects of energy by comparing MEPAs (6/15 MV 1:1 weighted) with 6 and 15 MV‐PA with the same arc lengths and optimization parameters, by eliminating the heuristic weighting scheme. This study showed that MEPAs (6/15 MV 1:1 weighted) improved CN, reduced doses to bladder and rectum by covering lower volume of OARs at all dose levels, and lowered MUs, but increased doses to the femoral heads compared to 6 and 15 MV‐PA. The results of Part 2 of this study are in agreement with the only previous study on mixed energy VMAT technique,^24^ which compared dual arcs mixed energy VMAT plans (one energy per one arc) with a single‐energy dual arcs plans for prostate cases involving seminal vesicles and lymph nodes. Our study involved comparison of a full arc split into MEPAs, MEPAs (6/15 MV 1:1 weighted), with a single‐energy partial‐arc plans (6 and 15 MV‐PA) for prostate only. Furthermore, for prostate cancer patients with AP separation greater than 21 cm, the higher energy (10 MV) plans were reported to be superior in sparing OARs and lowering monitor units compared to lower energy (6 MV) plans.^25^

In assessing clinical importance, it has been previously reported that rectum volume receiving ≤30 Gy reduced the incidence of several types of patient‐reported late rectal toxicities by 10%–18%.^26^ MEPAs (6/15 MV 1:2 weighted) reduced V_30Gy_ by 9% compared to 6 and 15 MV‐SA (Table 5). The rectum volume receiving ≥60 Gy is associated with late rectal complication,^27^ MEPAs (6/15 MV 1:1 weighted) technique reduced V_60Gy_ by 6% compared to both 6 and 15 MV‐PA techniques (Table 8). It has been reported that late rectal complications occurred in 3/7 patients and 4/7 patients when a dose of 70 Gy or more was delivered to at least 7% and 3% of the rectal volume, respectively.^28^ It should be noted that the V_70Gy_ ranged from 6% to 7% in Part 1 (Table 5) and 8% to 11% in Part 2 of this study (Table 8). In comparison to 6 and 15 MV‐SA, possible occurrences of post‐EBRT rectal complications might be reduced by MEPAs (6/15 MV 1:2 weighted) as it only delivers 1/3 of the PD with bladder and rectum being in direct path of the beam. Complications in femoral heads such as fractures and necrosis can be kept to less than 5% if the mean dose to <50 Gy to limit.^29^ Though MEPA (6/15MV 1:2 weighted) delivers 2/3 of the PD from lateral arcs, the mean dose to the femoral heads were well below 20 Gy. According to Cefaro et al., the likelihood of a fracture of the femoral heads is greater than 5% when maximum dose to the femoral heads exceeds 40–45 Gy.^30^ The maximum dose deposited to the left and right femur by MEPA (6/15 MV 1:2 weighted) technique was 38 and 41 Gy, respectively. This is due to the greater proportion of PD delivered from lateral direction. In future studies, the potential of MEPAs technique can further be improved by optimizing the dose weighting factor for each energy in MEPAs plans. The dose‐volume specifications for bladder complications are not as well studied as for rectum. Vargas et al. have reported that reductions in the low doses area for bladder have been associated with lower long‐term urinary side effects.^31^ MEPA (6/15 MV 1:2 weighted) reduced the volume covered by 20 and 30 Gy by 2% compared 6 and 15 MV‐SA.

Furthermore, it has been reported that dose ≥78 Gy to 50% of the bladder volume results in the development of GU complications,^32^ which was not exceeded by any of the plans in this study. It is important to note here that maximum dose to bladder exceeded 65 Gy, especially in the overlapping region of bladder and the PTV, which involves the risk of Grade 3 toxicity as a late response.^29^ However, this was mainly due to not including maximum bladder dose constraints during optimization for any of the three techniques. This was because it is considered a strict constraint — required to be achieved by every single voxel of a structure, which, in turn, would require us to change the optimization parameters and optimize the plans individually. Instead, the goal was to optimize all the plans with a fixed optimization setup to highlight superiority among different techniques. In terms of prostate motion, a greater prostate motion has been reported to occur in anterior and posterior direction than lateral direction.^33^ Furthermore, it has been demonstrated that intrafraction prostate motion from breathing is a major cause of prostate positional variation.^34^ Although lower MUs would reduce the total treatment time resulting in lower probability of such organ motion, the total treatment time for MEPAs technique, regardless of the lower MUs, may not be reduced significantly as two different energies need to be moded up at the console for each treatment fraction.

Historically, patient separation in anterior posterior direction greater than 20 cm were considered as a threshold for using higher photon energy,^35^ the mean AP separation in our study was ~23 cm. The rationale behind using the lowest clinical range (6 MV) to the highest clinical range (15 MV) was to exploit the maximum difference in dose deposition. Both MEPAs techniques in this study involved 15 MV, which raises a question of additional dose deposited by photo‐neutrons produced in the linac head. This may be of some concern for MEPA (6/15 MV 1:2 weighted) technique as 2/3 of the PD is delivered by 15 MV beam. One study on the measurement of photo‐neutron dose at isocenter from an 18 MV linac showed that the total neutron equivalent dose is two to three orders of magnitude smaller than the photon dose delivered to the patient.^36^ Nonetheless the amount of neutron dose in the vicinity of the patient should not be neglected, which is one of the limitations of this study. Therefore, prior to clinically employing MEPA with 15 MV and higher, additional risks of secondary cancers due to photo‐neutrons should be considered. Furthermore, mixed energies VMAT involving higher energy would not be recommended for patients with pacemakers as it can result in the device malfunction.^37^ Since the neutron production for higher energy (>10 MV) in FFF mode is reduced as much as 70%,^29^ similar mixed energy technique for flattening filter free (FFF) modality would be an interesting topic for future investigation, though clinical use of FFF modality is currently limited to ≤10 MV.

Another limitation of our work is the same set of optimization parameters including priority weighting factors used for all the patients in Part 1 and 2 of this study. Our rationale behind maintaining same parameter set was to ensure that the differences were only due to energy and dose weighting selection in Part 1, and energy selection in Part 2 of this study. This approach permitted reliable comparison to justify a superior treatment planning technique for each part of this study. However, in practice, the optimization parameters of MEPAs plans specific to individual patients and corresponding treatment planning goals can further improve quality of MEPAs plans including reductions in maximum doses to bladder and femoral heads.

In terms of implications of MEPAs technique to clinical work‐flow, determining an ideal proportion of PD dedicated to each of the selected energies would be crucial to achieve desired dosimetric outcome. However, given that current TPS does not allow the optimization of proportion of PD dedicated to each energy for a mixed energy VMAT plan, determining an ideal proportion of PD dedicated to each energy in MEPAs plan would require a trial and error process, especially with different combination of energies and dose weighting factors. For instance, MEPAs can also be used in combination of 6 and 10 MV, which has less concerns of production of secondary neutrons in comparison to the combination of energies used in this study, 6 and 15 MV. We used the lowest and highest clinical MV range to exploit the maximum difference in dose deposition. Nevertheless, once established, MEPAs can easily be implemented for post optimization stages (i.e, patient specific QA) as the patient specific QA for MEPAs plans can be performed similarly to that of a single‐energy VMAT plans. This study was based on comparisons of TPS generated dosimetric outcomes. Any quality assurance of these plans was not considered as it was beyond the scope of this work. Finally, the radiobiological impact of any of the techniques used in this study was not investigated.

The TPS used in this study (RapidArc™, Eclipse, Palo Alto, CA, USA) does not allow optimization of a single plan with two different energies. Therefore, a composite plan was generated by summing a lower energy and a higher energy plan. Beside the TPS used in this study, the RayStation™ (Raysearch Laboratories, Stockholm, Sweden) and the Monaco™ (Elekta, Stockholm, Sweden) are two major treatment planning systems that are currently being used to optimize VMAT treatment plans. However, to our knowledge, no current treatment planning system, including the one used in this study, allows simultaneous optimization of two different energies. The current study, thus, involved the manual selection of dose weighting per energy to achieve the desire dosimetric outcome. An algorithm that simultaneously optimizes for both energies is necessary as it will generate a plan with an optimal proportion of PD dedicated to each energy, which, in turn, will further improve the quality of a mixed energy VMAT plan. While it was beyond the scope of this work to investigate the most suitable TPS for MEPAs technique, it would be interesting to investigate MEPAs on RayStation™, which utilizes multicriteria optimization where the user navigates through many pareto optimal plans to arrive at a plan with desired dosimetric tradeoffs. However, the dosimetric comparisons between two plans may not be suitable for RayStation™ as due to selection of best possible tradeoff between different dose‐volume objectives of various structures, the parameters may not remain same in the two plans.

## 5 CONCLUSIONS

This study investigated the potential scope of using MEPAs VMAT technique to treat prostate cancer compared to single‐energy VMAT techniques. In Part 1 of this study, MEPAs (6/15 MV 1:2 weighted) plans were found to be superior in sparing bladder and rectum, but resulting in slightly reduced target homogeneity compared to either 6 and 15 MV‐SA plans. In Part 2 of this study, the impact of multiple energies alone was investigated by equally weighting both 6 and 15 MV in MEPAs (6/15 MV 1:1 weighted) and comparing with single‐energy partial arcs (6 and 15 MV‐PA). MEPAs (6/15 MV 1:1 weighted) plans resulted in improved target dose conformity and, lower doses to bladder and rectum compared to 6 and 15 MV‐PA. In both parts, however, mixed energy VMAT plans increased doses to femoral heads compared to single‐energy VMAT plans.

## CONFLICT OF INTEREST

None.
